# Genomewide Analysis of Clp1 Function in Transcription in Budding Yeast

**DOI:** 10.1038/s41598-017-07062-6

**Published:** 2017-07-31

**Authors:** Nadra Al-Husini, Ali Sharifi, Seyed Ahmad Mousavi, Hamidreza Chitsaz, Athar Ansari

**Affiliations:** 1 Department of Biological Science, Wayne State University, Detroit, MI 48202 USA; 2 Department of Computer Science, Colorado State University, Fort Collins, CO 80523 USA; 3 Department of Stem Cells and Developmental Biology, Cell Science Research Center, Royan Institute for Stem Cell Biology and Technology, ACECR, Tehran, Iran; 4 Department of Computer Engineering, Sharif University of Technology, Tehran, Iran

## Abstract

In budding yeast, the 3′ end processing of mRNA and the coupled termination of transcription by RNAPII requires the CF IA complex. We have earlier demonstrated a role for the Clp1 subunit of this complex in termination and promoter-associated transcription of *CHA1*. To assess the generality of the observed function of Clp1 in transcription, we tested the effect of Clp1 on transcription on a genomewide scale using the Global Run-On-Seq (GRO-Seq) approach. GRO-Seq analysis showed the polymerase reading through the termination signal in the downstream region of highly transcribed genes in a temperature-sensitive mutant of Clp1 at elevated temperature. No such terminator readthrough was observed in the mutant at the permissive temperature. The poly(A)-independent termination of transcription of snoRNAs, however, remained unaffected in the absence of Clp1 activity. These results strongly suggest a role for Clp1 in poly(A)-coupled termination of transcription. Furthermore, the density of antisense transcribing polymerase upstream of the promoter region exhibited an increase in the absence of Clp1 activity, thus implicating Clp1 in promoter directionality. The overall conclusion of these results is that Clp1 plays a general role in poly(A)-coupled termination of RNAPII transcription and in enhancing promoter directionality in budding yeast.

## Introduction

**Figure 1:**
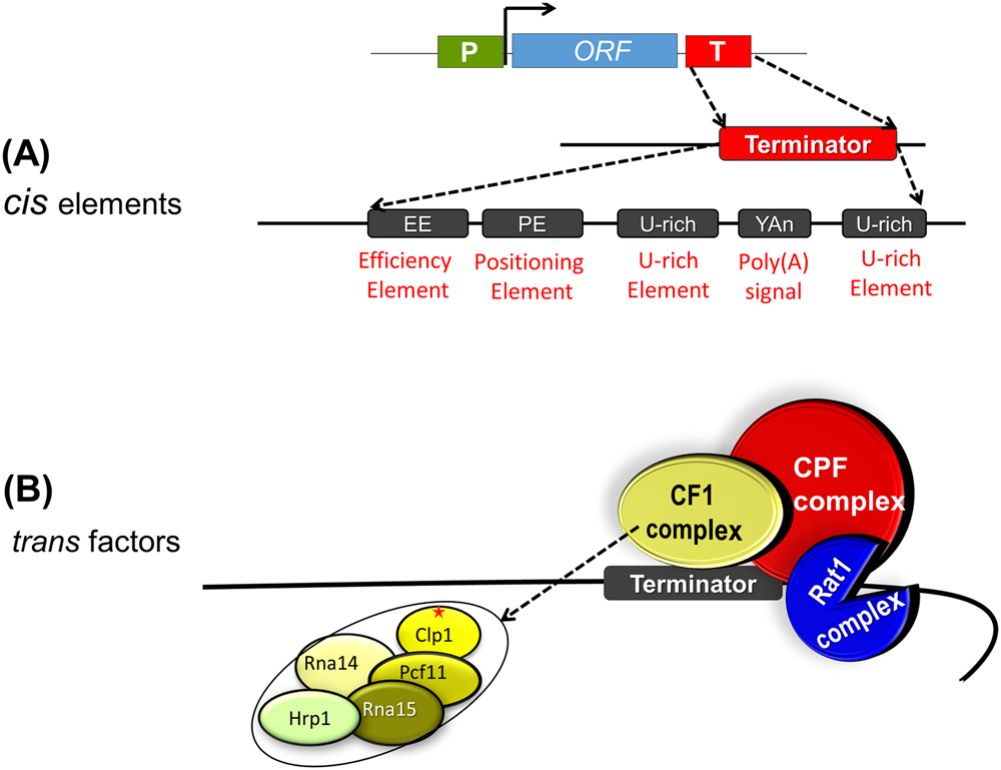
Termination of transcription in yeast requires DNA elements located near the 3′ end of genes and accessory protein factors. (**A**) *Cis*-acting DNA elements required for termination of transcription in budding yeast. (**B**) CF 1, CPF and Rat1 are three multisubunit protein complexes required for termination of transcription in budding yeast. CF1 subunit Clp1 shown with an asterisk is the focus of this investigation.

The CF IA 3′ end processing complex of budding yeast is composed of four subunits: Rna14, Rna15, Pcf11 and Clp1 as shown in Fig. 1B ^1–3^. The complex has been implicated in cleavage-polyadenylation and coupled termination of transcription^4^. The termination function of the CF IA complex was demonstrated using a nuclear run-on assay that revealed the readthrough of RNAPII beyond the poly(A) site in the mutants of all four CF IA subunits^4, 5^. The transcription readthrough phenotype, however, was demonstrated for just a few selected yeast genes. ChIP analysis identified that the whole CF IA complex is localized at the 3′ end of selected genes in accordance with its role in 3′ end processing and termination of transcription^5, 6^. Genomewide analysis found Pcf11 crosslinked to the 3′ end of a majority of transcriptionally active yeast genes^7^. These observations suggests that the CF IA complex could be playing a general role in the termination of transcription in budding yeast. More direct evidence, however, was needed in support of a role for the CF IA complex in the termination of transcription on a genomewide scale and to firmly establish it as a general termination factor.

A vast majority of RNAPII-transcribed genes in yeast and higher eukaryotes exhibit antisense transcription initiating from both their 5′ as well as the 3′ ends^8, 9^. In yeast, the 5′ end initiated antisense transcripts are rapidly degraded by the RNA surveillance machinery of the cell and are therefore referred to as ‘cryptic unstable transcripts’ (CUTs)^10^. In contrast, the 3′ end initiated antisense transcripts are stable and belong to the category of ‘stable unannotated transcripts’ (SUTs)^8^. At least 50% of SUTs in budding yeast are 3′ end initiated antisense transcripts. A number of genes in yeast and mammalian cells exhibit 3′ end initiated antisense transcription under repressed conditions. The *GAL10* gene of budding yeast, for example, exhibits robust sense transcription in the presence of galactose^11, 12^. Upon shifting of cells to glucose containing medium, however, sense transcription is almost completely inhibited, and the 3′ end initiated antisense transcription predominates. It has been proposed that when the 3′ end initiated antisense transcript reaches the promoter end of the gene, it may adversely affect initiation/reinitiation either through transcriptional interference or through histone modifications^13–15^. Whether 3′ end initiated antisense transcription is a general feature of termination defective mutants is not clear.

Since the CF IA complex is involved in the cleavage-polyadenylation of mRNA and termination of transcription, it is expected to be present near the 3′ end of genes. It was, however, intriguing to find all four subunits of the CF IA complex occupying the 5′ end of genes as well^6^. Furthermore, the Ssu72 subunit of the CPF complex, which is also linked to 3′ end processing and termination of transcription in budding yeast, has been localized to the promoter end of genes^16, 17^. The presence of termination factors towards the 5′ end of genes is an evolutionarily conserved feature as a number of 3′ end processing/termination factors of higher eukaryotes also have been found crosslinked to the distal ends of genes^18^. The promoter occupancy of termination factors remained an enigma until recently when it was demonstrated that these factors provide directionality to transcription by limiting divergent antisense transcription initiating from the promoter region^5, 10, 18–20^. The nucleosome free region located at the 5′ end of a majority of protein coding genes in yeast and mammals contains two juxtaposed unidirectional promoters^21–25^. The transcription from these promoters initiates in both the downstream sense direction as well as the upstream antisense direction. The downstream sense transcription of the coding region produces mRNA, while the upstream antisense transcription produces upstream antisense RNA (uaRNA)^10, 19^. Transcription in the upstream antisense direction is terminated within a few hundred bases from the transcription start site by the termination factors residing in the promoter region, while productive transcription in the sense direction is allowed to proceed until the polymerase reaches the 3′ end of the gene^10, 18–21, 26^. Thus, promoter linked termination factors provide directionality to the inherently bidirectional eukaryotic promoter region by limiting synthesis of uaRNA. It has been demonstrated that gene looping plays a crucial role in the termination of uaRNA synthesis in budding yeast^5, 10^. We recently showed that the CF IA complex is involved in providing transcription directionality during transcription of selected yeast genes^5^. Whether the CF IA complex has a general role in conferring promoter directionality during RNAPII-mediated transcription, however, needed further investigation.

Using a temperature-sensitive mutant of Clp1, we recently demonstrated the role of the CF IA complex in termination of transcription as well as in the promoter directionality of the *CHA1* gene in budding yeast^5^. Here we extend these studies to show that the CF IA complex has a global role in the poly(A)-dependent termination of transcription. Employing the GRO-Seq approach, we further showed that the CF IA complex also influence promoter directionality of subset of yeast genes. How the CF 1A complex affects promoter-associated transcription, however, remains to be elucidated.

## Results

### Clp1 is required for the termination of transcription on a genomewide scale

A combination of biochemical and genetic approaches has identified two macromolecular complexes called CF I and CPF being essential for both 3′ end processing and poly(A)-dependent termination of transcription in budding yeast (Fig. 1)^2, 27, 28^. In addition, the Rat1 complex is also required to accomplish the termination step. When termination is defective, RNAPII does not dissociate from the template beyond the poly(A) site, but reads through the poly(A) termination signal^2, 29^. This transcription readthrough phenotype is characteristic of defective termination, and has been widely used to determine the involvement of a factor in the termination of transcription. A reliable approach used for detecting the terminator readthrough phenotype is the nuclear run-on (NRO) assay^4^. This assay measures the density of transcriptionally active polymerase over a specific genomic locus. The genomewide version of this assay, called ‘Global Run-On-Seq (GRO-Seq), overcomes the limitations in the NRO assay in genome coverage and the strand specificity^21^. In GRO-Seq, the newly synthesized transcripts incorporate BrUTP, which allow for the affinity purification of these nascent transcripts on an anti-BrUTP column. The resultant nascent RNAs are subjected to high throughput sequencing. The outcome of GRO-Seq is a snapshot of the position as well as the density of the actively engaged polymerase in a strand specific manner.

**Figure 2:**
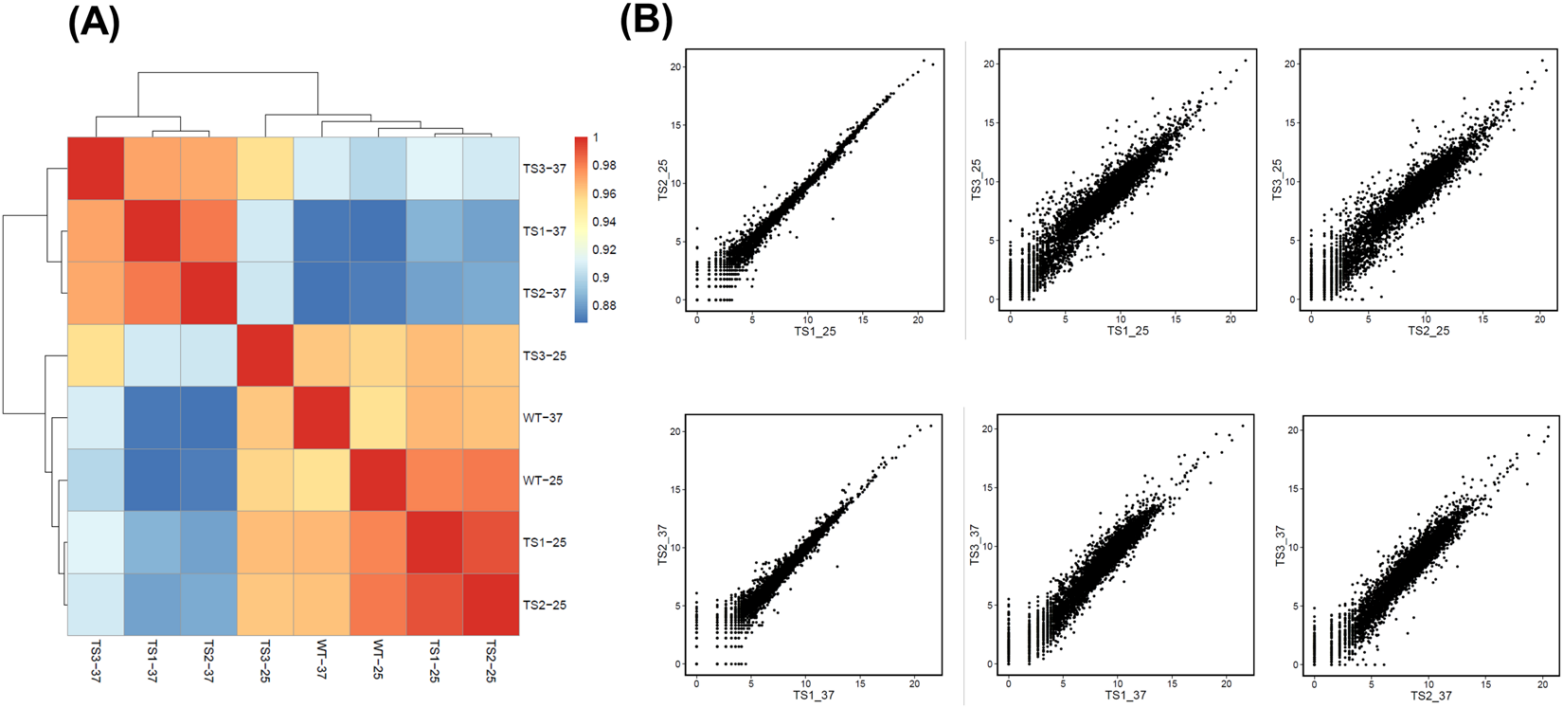
GRO-Seq experiments performed in triplicates in *clp1* mutant exhibit high degree of correlation. (**A**) Heatmap of pairwise Pearson Correlation Coefficients among all samples. (**B**) Scatter plots of indicated pair of samples.

To assess the role of the CF IA subunit Clp1 in transcription on a genomewide scale, we used the GRO-Seq approach. Comparing the GRO-Seq maps of the temperature-sensitive mutant of Clp1 and the isogenic wild type strain at the permissive (25 °C) and non-permissive (37 °C) temperatures of the mutant can reveal if Clp1 is a universal termination factor like the general transcription factors, or its role in termination is restricted to a subset of genes. Accordingly, we performed GRO-Seq analysis in the Clp1 mutant and the isogenic wild type strains in the cells grown at 25 °C and 37 °C. The experiments with the Clp1 mutant were performed in triplicates named TS-1, TS-2 and TS-3. The number of transcriptionally active genes was determined by using an experimentally determined threshold of 5 reads per kilo base. We found that ~5500 of the 6693 annotated ORFs (~82%) in the Saccharomyces Genome Database (SGD) were expressed as tags above the background. The heatmap of pairwise Pearson Correlation Coefficients among all samples shows that all TS-37 samples (TS1-37, TS2-37 and TS3-37) are clustered together, while TS-25 (TS1-25, TS2-25 and TS3-25), WT-25 and WT-37 samples are clustered together (Fig. 2A,B and Supplemental Fig. 1).

**Figure 3:**
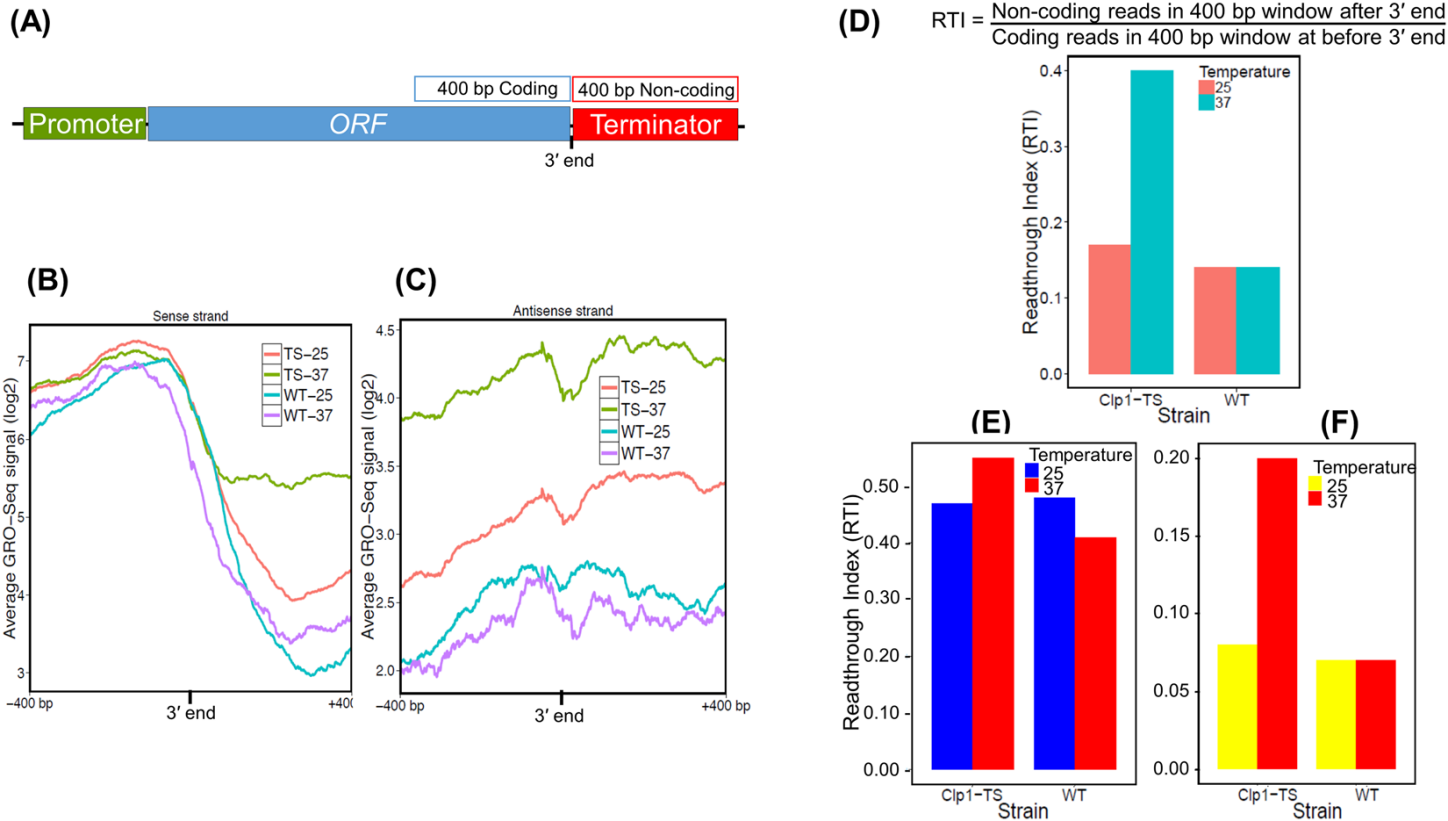
Alignment of GRO-Seq reads to the 3′ end in *clp1* mutant and the isogenic wild type strain revealed a role for Clp1 in general termination of transcription in budding yeast. (**A**) Schematic depiction of a gene showing the window of 400 bp coding and non-coding regions near the 3′ end of genes where the GRO-Seq reads were aligned. (**B**) GRO-Seq reads aligned to the 400 bp window flanking the 3′ end of genes in sense direction in *clp1-ts* mutant and the wild type strains at 25 °C and 37 °C. (**C**) GRO-Seq reads aligned to the 400 bp windows flanking the 3′ end of genes in antisense direction in *clp1-ts* mutant and the wild type strains at the indicated temperatures. (**D**) Readthrough Index (RTI) was calculated as indicated in *clp1-ts* mutant and the wild type strains at 25 °C and 37 °C. (**E**) RTI plots of genes with low transcription activity (RPKM value of less than 24.22), and (**F**) of those with high transcription activity (RPKM value more than 24.22) in *clp1-ts* mutant and the wild type strains at 25 °C and 37 °C.

To investigate the role of Clp1 in the termination of transcription, we mapped GRO-Seq reads to the annotated 3′ end of genes. Throughout this manuscript, the 5′ end is the start of the open reading frame, while the 3′ end is the end of the ORF of a gene. For this analysis, we removed all those genes whose next neighboring gene from the 5′ and 3′ end was less than 400 bp away. This was done because of the compact nature of the yeast genome, which often results in the terminator region of a gene overlapping with the promoter or terminator elements of the neighboring gene. We therefore restricted our analysis to 1038 genes whose 5′ and 3′ ends were at least 400 bp away from the neighboring ORF. All these 1038 genes were not transcriptionally active in the mutant and the wild type cells. The number of transcriptionally active genes varied from 664 to 844 as shown in Supplemental Table 1. We compared the transcription in the sense direction near the 3′ end of genes in the mutant and isogenic wild type strains at 25 °C and 37 °C. The wild type strain exhibited a distinct peak of activity towards the 3′ end of genes (Fig. 3B). The peak was followed by a sharp drop off beyond the presumed polyadenylation signal. A similar peak and drop in the GRO-Seq signal near the 3′ end of genes was observed in the mutant strain at the permissive temperature (25 °C) (Fig. 3B). In contrast, there was no drop off, but an increase in the number of reads beyond the polyadenylation signal in the mutant at 37 °C compared to 25 °C (Fig. 3B). No such increase in the number of GRO-Seq signal was observed in the wild type cells at 37 °C (Fig. 3B). A logical conclusion of these results is that the polymerase was unable to read the poly(A) termination signal in the mutant at 37 °C leading to an enhanced readthrough signal in the 3′ downstream region of the gene.

**Figure 4:**
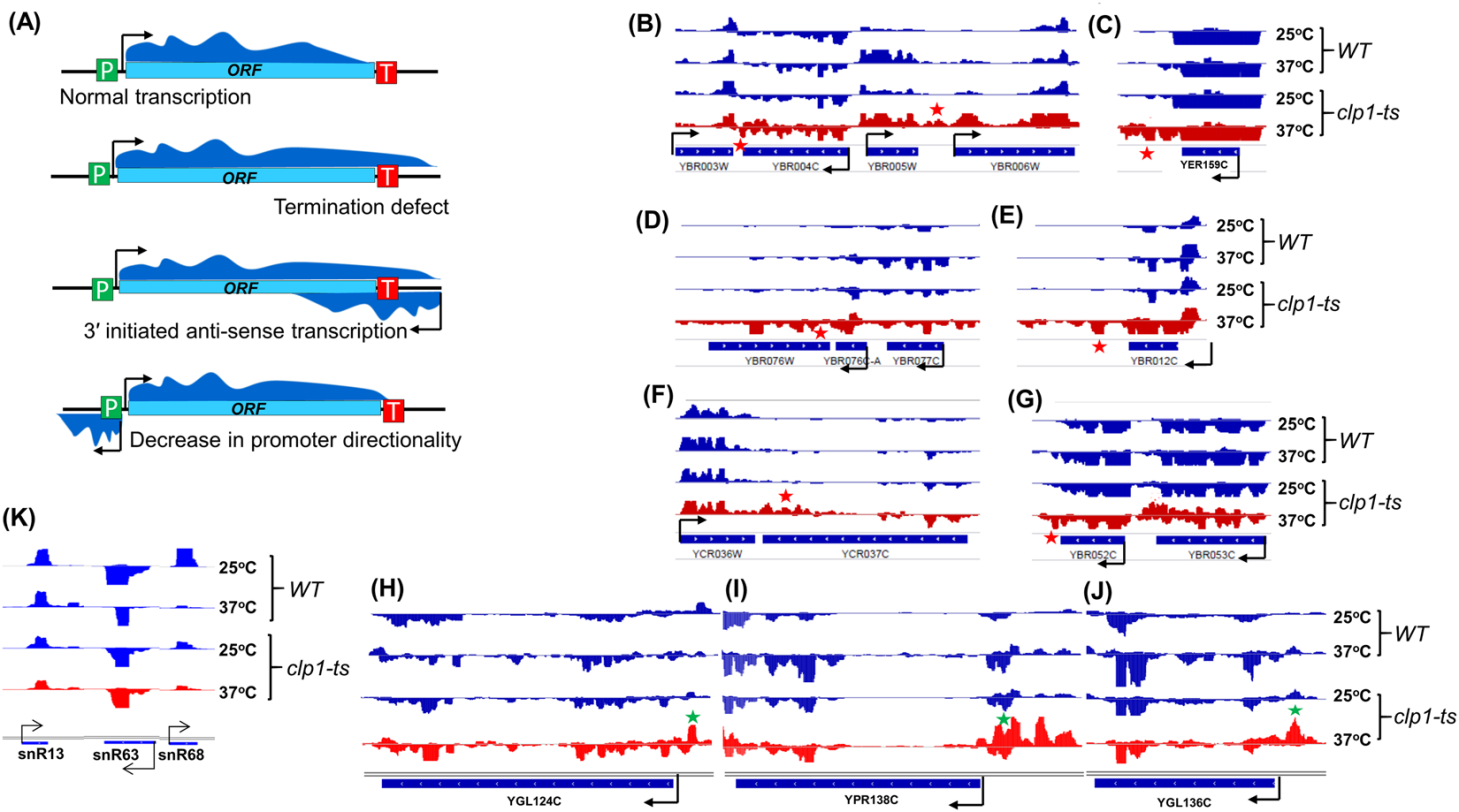
Snapshot of individual genes from GRO-Seq analysis. (**A**) Schematic depiction of a genes showing expected distribution of GRO-Seq reads on a gene when termination is normal or defective or when there is 3′ end initiated or 5′ end initiated antisense transcription. (**B**–**J**) GRO-Seq reads aligned to selected individual genes in sense and antisense direction in *clp1-ts* mutant and the wild type strains at 25 °C and 37 °C. Arrow indicates the transcription start site (TSS) and the direction of transcription. Name of each gene is indicated below. Red star shows transcription readthrough phenotype, while green star indicates promoter-associated upstream antisense transcription in the mutant at 37 °C. Scale of Y-axis in each gene snap shot is given in Supplemental Table 2. (**K**) GRO-Seq reads aligned to three snoRNA genes in sense and antisense direction in *clp1-ts* mutant and the wild type strains at 25 °C and 37 °C. Scale of Y-axis is given in Supplemental Table 2.

A number of gene specific examples are provided to illustrate the role of Clp1 in termination. These genes show an increase in GRO-Seq signal beyond their 3′ ends in the mutant at 37 °C (Fig. 4B–G). Since GRO-Seq approach has never been used before to study termination of transcription in budding yeast, we wanted to confirm the effectiveness of the approach to study the global transcription termination in yeast. We therefore examined transcription of two genes, which exhibited the termination defective phenotype in our GRO-Seq analysis, by strand-specific TRO assay. We selected *RFS-1* (YBR052C) and *GPI18* (YBR004C) genes, which exhibit the termination defect in the GRO-Seq analysis in clp1 mutant at 37 °C (Fig. 4G and B). The strand-specific TRO analysis of *RFS1* and *GPI18* revealed comparable results. Both genes exhibited terminator readthrough phenotype in the mutant at elevated temperature (Supplemental Fig. 2). These results corroborated the suitability of GRO-Seq approach in studying termination of transcription on a genomewide scale in yeast.

To further characterize the role of Clp1 in termination on a genomewide scale, we calculated the readthrough index (RTI) in the mutant and wild type cells grown at 25 °C and 37 °C. RTI was calculated by dividing the number of reads beyond the 3′ end of a genes with the number of reads before the 3′ end within the coding region. A termination defect will result in more GRO-Seq reads downstream of the 3′ end of a genes and therefore a higher RTI value. Accordingly, RTI value increased by about 2-fold in the mutant at 37 °C compared to at 25 °C (Fig. 3D). No such increase was observed in the wild type strain at elevated temperature (Fig. 3D). These results strongly suggested that Clp1 is required for termination of transcription by RNA polymerase II in budding yeast. It was, however, not clear from this analysis if Clp1 is required for termination of transcription of all RNA polymerase II-transcribed genes or its function is restricted to a subset of class II genes. To address the issue, we divided 664 transcriptionally active genes in WT-25 sample (see Supplemental Table 1) into two categories, one with high expression level (higher than median in WT-25) and the other with low expression level (lower than median in WT-25). The low expression group consisted of 355 genes, while the high expression group comprised of 309 genes. We calculated RTI values for both categories in the mutant and the wild type cells at 25 °C and 37 °C as described above. The genes with low transcription activity exhibited inefficient termination even in the wild type cells and exhibited only a marginal increase in RTI in the mutant at elevated temperature (Fig. 3E). In contrast, genes with higher expression level showed strong dependence on Clp1 for termination of transcription as their RTI value increased by nearly 2.5-fold in the absence of Clp1 function (Fig. 3F).

Next, we examined if Clp1 is required for poly(A)-dependent termination of transcription only, or for the poly(A)-independent termination as well. The transcription termination of the snoRNA genes occurs in a poly(A)-independent manner. Most of the genes coding for snoRNA are located in the crowded region of yeast genome. We, therefore, looked at the termination of three snoRNA genes, snR13, snR63 and snR68, which are separated from the neighboring genes by several hundred base pairs. No termination defect was observed for these three genes in the clp1 mutant at elevated temperature (Fig. 4K). These results suggest that the role of Clp1 may be restricted to the poly(A)-coupled termination of transcription by RNAII in budding yeast.

### Clp1 and 3′ initiated antisense transcription

A number of genes both in yeast and mammalian systems exhibit 3′ initiated antisense transcription upon defective termination. It has been shown that the 3′ initiated antisense transcription inhibits the recruitment of transcription machinery at the promoter region for initiation of transcription. We, therefore, examined the role of Clp1 in antisense transcription originating from the 3′ end of genes. We choose the same set of 1038 genes for this analysis whose 5′ and 3′ ends were at least 400 bp away from the neighboring gene. Alignment of GRO-Seq reads to the 3′ end of these genes revealed enhanced antisense transcription in the Clp1 mutant at 37 °C compared to at 25 °C (Fig. 3C). No 3′ end antisense transcript enhancement was observed in the wild type strain at 37 °C (Fig. 3C). It is, however, difficult to distinguish the 3′ initiated antisense transcription from the 5′ initiated antisense transcription of the neighboring downstream gene, and the readthrough transcription from the convergent downstream gene. The relationship between 3′ initiated antisense transcription and the sense transcription in the Clp1 mutant therefore requires a more thorough scrutiny.

### Role of Clp1 in promoter-associated transcription

**Figure 5:**
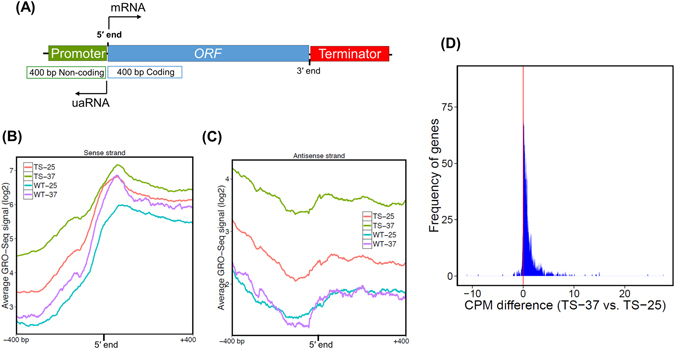
Alignment of GRO-Seq reads to the 5′ end suggests a role for Clp1 in promoter directionality of genes. (**A**) Schematic depiction of a gene showing the window of 400 bp coding and non-coding regions near the 5′ end of genes where the GRO-Seq reads were aligned. (**B**) GRO-Seq reads aligned to the 400 bp window flanking the 5′ end of genes in sense direction in *clp1-ts* mutant and the wild type strains at 25 °C and 37 °C. (**C**) GRO-Seq reads aligned to the 400 bp windows flanking the 5′ end of genes in antisense direction in *clp1-ts* mutant and the wild type strains at the indicated temperatures. (**D**) A histogram showing the number of genes exhibiting a decrease in promoter directionality upon shifting the mutant cells to non-permissive temperature. A plot of CPM difference in the 100 bp non-coding window upstream of the promoter was calculated in the mutant at 37 °C and 25 °C and plotted as a function of fraction of genes.

We have earlier showed that Clp1 plays a crucial role in promoter-associated transcription of *CHA1* by facilitating reinitiation and conferring promoter directionality^5^. We, therefore, examined if Clp1 is facilitating initiation/reinitiation of transcription on a genomewide scale. If Clp1 was facilitating initiation/reinitiation of transcription, we expected a decrease in the density of mRNA transcribing polymerase in the mutant at 37 °C. When we mapped GRO-Seq reads of the mutant and the wild type cells grown at 37 °C, to the annotated 5′ end of genes, there was no decrease in the GRO-Seq signal for sense transcribing polymerase in the mutant at elevated temperature (Fig. 5B). In fact, there was a slight increase in the density of mRNA-transcribing polymerase in the promoter-proximal 400 bp coding region in the mutant at 37 °C (Fig. 5B). A similar polymerase peak was observed in the promoter-proximal downstream region in the mutants of termination factors in mammalian cells^19^. These results argue against a role for Clp1 in recruitment of polymerase for initiation/reinitiation of transcription.

Next, we determined the role of Clp1 in transcription directionality. To determine the generality of Clp1 function in the termination of uaRNA synthesis, and thus the maintenance of promoter directionality, we mapped GRO-Seq reads in the mutant and the wild type cells grown at 25 °C and 37 °C, to the annotated 5′ end of genes. For this analysis, we chose the same set of 1038 genes whose 5′ and 3′ ends were at least 400 bp away from the neighboring gene. The GRO-Seq reads in antisense direction upstream of the 5′ end of genes represent uaRNA, while GRO-Seq reads in the promoter downstream sense direction represent mRNA (Fig. 5A). As described above, there was only a marginal increase in mRNA reads in 400 bp promoter downstream coding window both in the mutant and wild type cells at elevated temperature (Fig. 5B). In contrast, a two fold increase in the intensity of uaRNA signal was observed in the mutant at 37 °C compared to 25 °C in 400 bp promoter upstream non-coding window (Fig. 5C). No such increase was observed in the wild type cells at elevated temperature. Thus, there was a decrease in promoter directionality in the mutant at 37 °C. A number of gene specific examples are provided to illustrate the role of Clp1 in promoter directionality (Fig. 4H–J). These genes show an increase in GRO-Seq signal in the promoter-upstream antisense direction in the mutant at 37 °C.

To compute the number of genes exhibiting a decrease in promoter directionality in terms of enhanced uaRNA transcription in clp1 mutant at elevated temperature, we calculated ratio of GRO-Seq reads in 100 bp promoter upstream region in the antisense direction for individual genes in the mutant at 37 °C and 25 °C. We computed the difference in CPM (Count Per Million) reads in the mutant at the permissive and non-permissive temperatures as described in ‘Methods’ section. Our results indicate that of around 700 transcriptionally active genes analyzed in this study, 330 genes exhibited the CPM difference greater than +1 (in log2 scale, which means at least 2-fold higher uaRNA expression in the mutant at 37 °C), while only 13 genes had CPM difference less than −1 (Fig. 5D). These results clearly demonstrate that the uaRNA transcription significantly increased in the clp1 mutant at 37 °C for 330 genes (*p-*value < 1E-15, Wilcoxon signed rank test), while remaining approximately 60% of the transcriptionally active genes analyzed in this study did not exhibit any change in promoter directionality at elevated temperature. The overall conclusion of these results is that Clp1 influences the promoter directionality of nearly 40% of transcriptionally active genes analyzed in our study. These include both highly expressed and lowly expressed genes.

## Discussion

Of the three steps of the RNAPII transcription cycle (initiation, elongation and termination), initiation is the most well understood step. The general initiation factors are well characterized, and it is possible to perform initiation of transcription from a defined promoter using highly purified factors under *in vitro* conditions. In contrast, termination is the least understood step of the transcription cycle. The accessory factors required for termination are not thoroughly characterized. Consequently, termination of transcription under *in vitro* conditions using purified yeast factors has not been achieved so far in budding yeast. A thorough understanding of the process of termination by RNAPII necessitates identification of the general termination factors in line with the general transcription factors that are required for initiation of transcription. A number of approaches like PAR-CLIP, GRO-Seq, PRO-Seq, and Net-Seq can be used to determine the genomewide role of an identified factor in termination of transcription. All these approaches may give similar results, but there are subtle differences in the precise information they reveal. We used GRO-Seq approach because of its high resolution and sensitivity in detecting defective termination.

An important conclusion of the GRO-Seq analysis performed here is that Clp1 is required for termination of transcription of RNPII-transcribed genes in budding yeast. Our analysis further show that Clp1 is required for poly(A)-dependent termination of transcription of relatively highly expressed genes. Since there is no report of Clp1 acting alone or being a part of a complex other than CF IA, we extrapolate these results to propose that the whole CF IA complex, and not its individual components separately, is required for termination of transcription. Our results are corroborated by a recent finding that another CF 1 A subunit Pcf11 is required for termination of transcription on a genomewide scale^30^. A similar genomewide analysis was performed for CstF64, which is the mammalian homologue of another CF 1A subunit Rna15^19^. This analysis identified CstF64 as a general termination factor in mammalian RNAPII transcription cycle. Thus, the role of CF 1A complex and its mammalian counterpart in termination of transcription has been conserved during evolution. Besides CF 1A complex, termination of transcription in yeast requires CPF complex. It was recently demonstrated using PAR-CLIP approach that the CPF subunit Ysh1 is required for global termination of transcription^31^. The termination defective phenotype of Ysh1, however, was quite different from that of Clp1. Unlike Clp1, depletion of Ysh1 did not lead to readthrough transcription, but resulted in the accumulation of polymerase beyond the poly(A) site. These results suggested a role for Ysh1 in dissociating polymerase from the template beyond the poly(A) site. In contrast, CF 1A complex could be facilitating slowing down of polymerase near the poly(A) site, which is an important step in termination of transcription by RNAPII.

During transcription, the distal ends of a gene physically interact with each other forming a looped gene architecture^16, 32^. The proximity of the promoter and terminator regions in a gene loop allows promoter-bound factors to influence termination of transcription^6, 33–36^. The gene looping also invokes the possibility of termination factors affecting the promoter-associated transcription. Accordingly, it was demonstrated that Ssu72, which is a component of CPF termination complex, affects directionality of promoter-initiated transcription^10^. We also showed that Clp1 affects transcription directionality of *CHA1* ^5^. Clp1 is also required for gene looping of *CHA1* ^5^. The results of this genomewide analysis demonstrate that, apart from its role in termination, Clp1 also function in regulating promoter directionality of RNAPII-transcribed yeast genes. Clp1 suppresses the promoter-initiated upstream antisense transcription of 330 out of about 700 transcriptionally active genes used in this analysis. The promoter-initiated downstream antisense transcription either remained unaffected or exhibited a marginal increase for a majority of genes in the absence of Clp1 activity. Thus, the role of Clp1 in promoter directionality is similar to that of CPF subunit Ssu72^10^. CAF-1 complex, which is associated with chromatin assembly pathway, also affected promoter-initiated divergent non-coding transcription of yeast genes in a manner similar to Clp1^37^. Both Ssu72 and CAF-1 complex suppress promoter-initiated uaRNA transcription. The transcription of promoter-associated mRNA, however, either remains unaffected or exhibited a small increase in the absence of Ssu72 or CAF-1 activities. The NET-Seq analysis performed in mammalian cells likewise revealed the role of CstF64 and Xrn2 termination factors in transcription directionality^19^.

Overall conclusion of these results is that the CF 1A complex plays a role in poly(A)-coupled termination of transcription, and in limiting promoter-associated divergent antisense transcription on a genomewide scale in budding yeast. Not all RNAPII-transcribed yeast genes, however, are dependent on CF 1A complex for either termination of their transcription or promoter directionality. This is expected as there is an alternate pathway for termination of transcription in budding yeast that requires Nrd1-Nab3-Sen1 complex. Nrd1-dependent termination has been shown to affect termination of non-polyadenylated mRNA as well as uaRNA belonging to the category of CUTs. Is it gene looping that governs if termination of transcription and promoter directionality will be accomplished by the CF 1 A complex or the Nrd1 complex remains to be elucidated?

## Methods

### Yeast Strains

The yeast strains used in this study are BY4733 (*MATa his3Δ200 trp1Δ63 leu2Δ0 met15Δ0 ura3Δ0*) and clp1-769-5 (*MATa ura3Δ0 leu2Δ0 his3Δ1lys2Δ0can1Δ::LEU2-MFA1pr::His3 clp1ts::URA3*).

### GRO-Seq approach

GRO-Seq analysis was performed by the modification of method described in Core *et al*.^21^.

#### Step I: Preparation of permeabilized cells

The *clp1-769-5* mutant or the wild type cells were grown at 25 °C in 100 ml of YPD (Yeast extract-Peptone-Dextrose) medium to an OD_600_ of 0.4. The 100 ml culture was transferred to two 50 ml sterile tubes, and centrifuged at 1860 × g for 5 minutes. Cell pellets were resuspended in 50 ml of 25 °C or 37 °C prewarmed YPD medium, and grown at the permissive (25 °C) or non-permissive temperature (37 °C) for 2 hours. This normally takes cells to an OD_600_~0.8. Equal number of cells were pelleted by centrifugation at 1860 × g for 5 minute at 4 °C. Cell pellets were resuspended in 10 ml of ice-cold TMN buffer (10 mM Tris-HCl pH 7.5, 5 mM MgCl_2_, 100 mM NaCl), incubated in ice for 10 minutes, centrifuged at 1860 × g for 5 minutes, and then resuspended in 940 μl of DEPC (Diethylpyrocarbonate)-treated ice-cold water. Cells were permeabilized by incubating with 60 μl 10% sarkosyl at 4 °C for 25 minutes with gentle shaking. Permeabilized cells were spun down at 1200 × g for 6 minutes at 4 °C. The pellets were stored at 4 °C till all samples were ready for the next step.

#### Step II: Transcription run-on reaction and isolation of nascent RNA

Transcription run-on reaction was performed by the modification of methods described in Birse *et al*., (1997). Cell pellets from step I above were resuspended in 150 μl of transcription run-on reaction buffer (50 mM Tris-HCl pH 7.5, 100 mM KCl, 10 mM MgCl_2_, 2 mM DTT, 0.5 mM each of ATP, CTP, GTP and Brd-UTP) containing 5 μl of freshly added RNase Inhibitor (40 units/μl) and incubated at 30 °C for 5 minutes. The reaction was stopped by adding 500 μl of ice-cold Trizol reagent. About 250 μl of acid-washed glass beads were added to each tube. Cells were immediately lysed by vigorous shaking at 4 °C for 20 minutes. The lysate was transferred to a new 1.5 ml microfuge tube containing 500 μl of Trizol and 200 μl of chloroform. The tubes were shaken vigorously and centrifuged at 14000 rpm at 25 °C for 20 minutes. The supernatant was extracted with phenol-chloroform three times. Total RNA was precipitated by adding 3 volumes of ice-cold 100% ethanol, 2 μl of glycogen (20 mg/ml) and NaCl to a final concentration of 0.3 M followed by overnight incubation at −20 °C. The precipitated RNA was recovered by centrifugation at 16000 × g for 30 minutes at 4 °C. The RNA pellet was washed once with ice-cold 75% ethanol, air dried and resuspended in 55 μl of DEPC-treated water.

The extracted RNA was subjected to partial hydrolysis by incubating with 500 mM NaOH on ice for 20 minutes. The NaOH was neutralized by adding 30 μl of 1 M Tris-HCl (pH 6.6). Partially hydrolyzed RNA was purified using RNeasy kit (Qiagen) to remove any unincorporated nucleotides and to exchange buffer. RNA was eluted from the RNeasy column twice with 50 μl of DEPC treated water.

#### Step III: Immunopurification of Brd-UTP-labeled RNA

About 25 μl of anti-BrdU-antibody-conjugated agarose beads were washed three times with 500 μl of binding buffer (0.25x SSPE buffer, 1 mM EDTA, 0.05% Tween20, 37.5 mM NaCl). Beads were blocked by gently shaking with 500 μl of blocking buffer (485 μl binding buffer containing 5 μl of 10% polyvinylpyrrolidone and 10 μl of Ultrapure BSA) for two hours at 4 °C. The blocked beads were washed twice with 500 μl of binding buffer and then resuspended in 400 μl of the same buffer. The RNA from step II was incubated at 65 °C for 5 minutes to get rid of any secondary structure and immediately placed on ice for 2 minutes. The RNA was then bound to the processed anti-BrdU beads with gentle shaking for one hour at 4 °C. Beads were then washed sequentially with 500 μl of binding buffer, 500 μl of low salt buffer (0.2x SSPE, 1 mM EDTA, 0.05% Tween20) and 500 μl of high salt buffer (0.25x SSPE, 1 mM EDTA, 0.05% Tween-20, 100 mM NaCl) once each, followed by two washes of 500 μl of TET buffer (1xTE buffer, 0.05% Tween20). All washes were performed on nutator for three minutes each. All centrifugation steps between the washes were at 194 × g for 1 minute each, with incubation of tubes on ice for 30 seconds before removing wash buffer. The bound nascent RNA was eluted two times with 125 μl and one time with 250 μl of elution buffer (20 mM DTT, 150 mM NaCl, 50 mM Tris-HCl pH 7.5, 1 mM EDTA, 0.1% SDS) for a total elution time of 10 minutes in 42 °C water bath. The eluted RNA was precipitated with NaCl as described previously.

RNA pellet was washed with ice-cold 75% ethanol, air dried and resuspended in 12 μl of DEPC-treated water and quantified using a Nanodrop spectrophotometer.

#### Step IV: rRNA depletion, library preparation and sequencing

The nascent, purified RNA samples were first depleted of rRNA. The depletion of rRNA was performed by the Ribo-Zero™ Magnetic Kit (Epicenter) following manufacturer’s recommendations. GRO-Seq libraries were constructed using the ScriptSeq^TM^ complete Kit (Epicenter) following manufacturer’s instructions. DNA Sequencing was performed at the DNA Core Facility of Cornell University.

### Gene expression analysis and RTI calculation

We used Cutadapt^38^ for quality control of raw fastq files to trim adapters and remove low quality reads. All high quality fastq files were mapped to Saccharomyces genome (Saccer3, downloaded from UCSC database) using Tophat2^39^ (version 2.1.1, parameters -i 30 -I 800) and HTSeq.^40^ (version 0.6) for calculating read counts. Subsequently DESeq. 2 was performed for read count normalization. Then reads per kilobase of transcript per million (RPKM) mapped reads > = 5 RPKM was done for determining number of expressed genes^41^.

Pheatmap package is applied to generate pairwise Pearson correlation coefficients analysis on normalized count files, and ggplot2 package applied for creating pairwise scatter plot among all replicates.

To calculate ‘Read Through Index’ (RTI) score and expression difference of samples regards to their coding strand, again all high quality fastq files were mapped to three modified Gene Transfer format (GTF) files TSS, TTS and gene body regions of Saccer3 genome by Tophat2. In addition HTSeq and DESeq. 2 applied to obtain normalized read count. The output is used to calculate RTI score for each gene as shown in Fig. 3.

### RTI = 3′ end-400 bp noncoding-sense/3′ end-400 bp coding-sense

All scripts for calculating RTI were performed using R and ggplot2 package employed to generate density plot s and scatter plots.

### Calculating fraction of genes showing loss of promoter directionality in the mutant at elevated temperature

To determine the number of genes showing loss of promoter directionality due to increased uaRNA transcription in the mutant at 37 °C, we computed the count of reads in each sample in the 100 bp non-coding window upstream to the TSS. We performed cross-sample normalization by dividing the RAW count values to the total number of aligned reads (in millions) per sample to compute Count Per Million (CPM) reads. Then we computed the Log2 fold-change of mean CPM values between TS-37 vs. TS-25 samples and the sum of CPM values for TS-37 samples minus TS-25 samples. The CPM difference greater than +1 indicates more than 2-fold higher expression in TS-37 samples, while a CPM difference less than −1 shows more than 2-fold higher expression in TS-25 sample. The mean CPM difference value for all genes is +1.136, and the median value is +0.545.

### Accession number

The data present in this work was deposited in NCBI’s Gene Expression Omnibus (GEO) database under the accession number GSE71624.

## Electronic supplementary material

Supplemental Information
