# Supplementary material for: Genomewide Analysis of Clp1 Function in Transcription in Budding Yeast
Source: Sci Rep. 2017 Jul 31;7:6894. doi: 10.1038/s41598-017-07062-6 (PMC5537279; doi:10.1038/s41598-017-07062-6)
Supplement: Supplementary file 1 — Supplemental Information [file 41598_2017_7062_MOESM1_ESM.pdf]

Genomewide Analysis of Clp1 Function in Transcription in Budding Yeast

Nadra Alhusini[1], Ali Sharifi[2, 3, 4], Seyed Ahmad Mousavi[3], Hamidreza Chitsaz[2*] and Athar Ansari[1*]

[1]Department of Biological Science, Wayne State University, Detroit, MI 48202, USA

[2]Department of Computer Science, Colorado State University, Fort Collins, CO 80523, USA

[3]Department of Stem Cells and Developmental Biology , Cell Science Research Center, Royan Institute for Stem Cell Biology and Technology, ACECR , Tehran , Iran

[4]Department of Computer Engineering, Sharif University of Technology, Tehran, Iran

[*]Correspondence should be addressed to AA (email: ansari@biology.biosci.wayne.edu) or HC (email: chitsaz@chitsazlab.org)

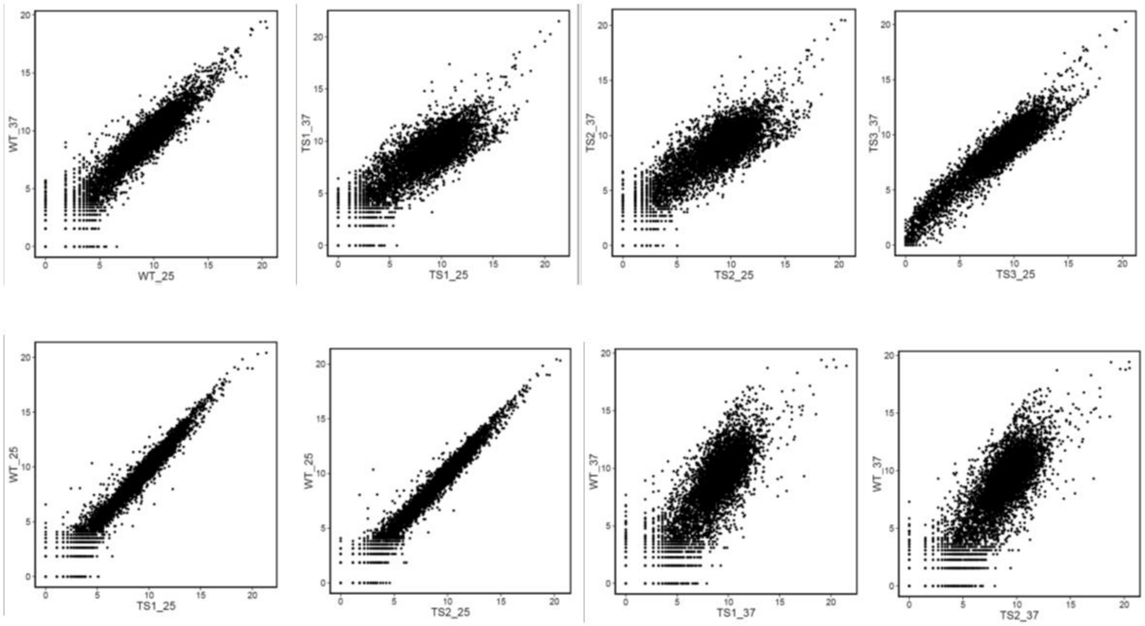

Supplemental Figure 1. GRO-Seq experiments performed in triplicates in *clp1* mutant.

Scatter plots of indicated pair of samples.

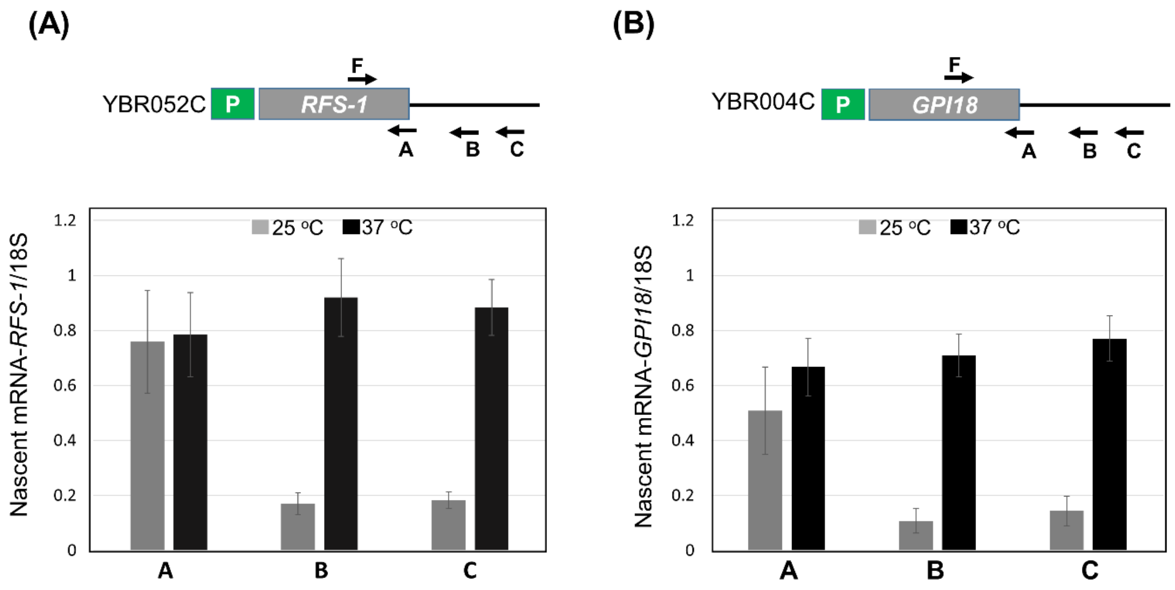

Supplemental Figure 2. Strand-specific TRO assay showing transcription termination defect in *FFS-1* and *GPI18* genes in *clp1* mutant at 37°C. Strand-specific TRO assay was performed for *RFS-1* (YBR052C) and *GPI18* (YBR004C) genes following the protocol described in Medler and Ansari (2015) in clp1 mutant at 25°C (grey bar) and 37°C (black bar). The results were compared with GRO-Seq results for these two genes shown in Fig. 4G and 4B. A, B and C are the primers used for cDNA synthesis. cDNA was PCR amplified by primer pairs F-A, F-B and F-C.

Supplemental Table 1. Transcriptionally active genes in different samples.

Number of transcriptionally active genes in TS-25 triplicates (TS1-25, TS2-25 and TS3-25), TS-37 triplicates (TS1-37, TS2-37 and TS3-37), WT-25 and WT-37 samples.

Sample	No. of transcriptionally active genes
TS1-25	699
TS2-25	704
TS3-25	792
TS1-37	844
TS1-37	843
TS1-37	846
WT-25	664
WT-37	738

Supplemental Table 2. Scale used in gene snapshots in Fig. 4.

Y-axis represents the expression of the gene in terms of terms of number of observed GRO-Seq reads.

Figure no.	Y-axis scale minimum	Y-axis scale maximum
4B	-1000	+1000
4C	-6000	+6000
4D	-800	+800
4E	-5000	+5000
4F	-1500	+5000
4G	-2000	+2000
4H	-500	+500
4I	-1500	+1500
4J	-1000	+1000
4K (snR13)	-120000	+120000
4K (snR63)	-90000	+90000
4K (snR168)	-90000	+90000
